# Myocardial Minimal Damage After Rapid Ventricular Pacing – the prospective randomized multicentre MyDate-Trial

**DOI:** 10.1038/s41598-020-61625-8

**Published:** 2020-03-16

**Authors:** Verena Semmler, Clara Deutschmann, Bernhard Haller, Carsten Lennerz, Amir Brkic, Christian Grebmer, Patrick Blazek, Severin Weigand, Martin Karch, Sonia Busch, Christof Kolb

**Affiliations:** 1 Deutsches Herzzentrum München, Klinik für Herz- und Kreislauferkrankungen, Abteilung Elektrophysiologie, Technische Universität München, Munich, Germany; 2 Klinikum rechts der Isar, Institut für Medizinische Informatik, Statistik und Epidemiologie, Fakultät für Medizin, Technische Universität München, Munich, Germany; 3 DZHK (German Centre for Cardiovascular Research), partner site Munich Heart Alliance, Munich, Germany; 4 Herz- und Gefäßzentrum Oberallgäu-Kempten, Klinikum Kempten, Kempten, Germany; 5 Klinikum Coburg, Abteilung für Kardiologie und Angiologie, Coburg, Germany

**Keywords:** Cardiac device therapy, Diagnostic markers

## Abstract

Therapy of choice for the primary and secondary prevention of sudden cardiac death is the implantation of an implantable cardioverter defibrillator (ICD). Whereas appropriate and inappropriate ICD shocks lead to myocardial microdamage, this is not known for antitachycardia pacing (ATP). In total, 150 ICD recipients (66 ± 12 years, 81.3% male, 93.3% primary prevention, 30.0% resynchronization therapy) were randomly assigned to an ICD implantation with or without intraoperative ATP. In the group with ATP, the pacing maneuver was performed twice, each time applying 8 impulses à 6 Volt x 1.0 milliseconds to the myocardium. High sensitive Troponin T (hsTnT) levels were determined prior to the implantation and thereafter. There was no significant difference in the release of hsTnT between the two randomization groups (delta TnT without ATP in median 0.010 ng/ml [min. −0.016 ng/ml–max. 0.075 ng/ml] vs. with ATP in median 0.013 ng/ml [min. −0.005–0.287 ng/ml], p = 0.323). Setting a hsTnT cutoff of 0.059 ng/dl as a regularly augmented postoperative hsTnT level, no relevant difference between the two groups regarding the postoperative hsTnT levels above this cutoff could be identified (without ATP n = 10 [14.7%] vs. with ATP n = 16 [21.9%], p = 0.287). There was no significant difference in the release of high sensitive Troponin between patients without intraoperative ATP compared to those with intraoperative ATP. Hence, antitachycardia pacing does not seem to cause significant myocardial microdamage. This may further support its use as a painless and efficient method to terminate ventricular tachycardia in high-risk patients.

## Introduction

In addition to the optimal medical and interventional therapy of the underlying disease, the treatment of choice for the primary and secondary prevention of sudden cardiac death in high-risk patients is the implantation of an implantable cardioverter defibrillator (ICD). For the termination of ventricular arrhythmias, the ICD system provides two modalities: overdrive pacing (antitachycardia pacing [ATP]) and cardioversion or defibrillation with an ICD shock. ATP is well known for its high efficiency in the termination of ventricular arrhythmias, at the same time being favored for its painlessness and battery saving behavior^1,2^. Therefore, it is well established in ICD therapy and usually programmed as the first therapy option. Except for possible acceleration of ventricular tachycardia, relevant side effects of ATP have not yet been found^1,2^. However, going along with the discussion whether ICD therapies themselves lead to an increased morbidity and mortality or whether the progression of the underlying disease is responsible for an adverse prognosis, the prospective multicenter MADIT-RIT study raised the question whether the ATP therapy itself increases the risk for an unfavorable long-term outcome in ICD patients^3^. Randomizing patients to different programming strategies to avoid early ICD intervention and reduce the number of inappropriate ICD therapies, the reduction of ATP seemed to reduce mortality in this population. Several studies have addressed this topic recently and the discussion of whether or not ICD therapies themselves might be harmful is ongoing. It has been shown earlier that ICD shocks can cause myocardial microdamage^4,5^. Whether ATP is associated with a similar myocardial damage is unclear and has not yet been studied thoroughly.

The aim of the MyDate (Myocardial Minimal Damage after rapid ventricular pacing) trial was therefore to characterize changes in levels of cardiac enzymes after rapid ventricular pacing, mimicking antitachycardia pacing, to assess whether this type of ICD therapy can cause myocardial damage.

## Methods

The MyDate trial was a prospective, randomized, multi-center trial (clinicaltrials.gov identifier: NCT02362802, date of first registration 13/02/2015). The study protocol was approved by the ethics committee of the Technical University of Munich (Ehtikkomission der Technischen Universität München) as leading ethics committee for the Deutsches Herzzentrum München, as well as approved by the ethics committees of the participating centers and complied with the conditions laid out by the Declaration of Helsinki. All patients gave their written informed consent prior to study inclusion.

### Study population

Patients scheduled for ICD implantation for primary or secondary prevention of sudden cardiac death and/or cardiac resynchronization therapy were included in the study, if the apical lead position and a left pectoral implantation site was intended. To keep confounding factors concerning the device and the energy applied to the myocardium low, all included patients received a Sorin device. The manufacturer of the leads was left to the implanting physicians’ choice.

Patients were excluded from the study, if they met one of the following criteria:

- Resuscitation or heart surgery or acute coronary syndrome or acute myocardial infarction or revascularization of coronary arteries or external cardioversion or ablation within four weeks prior to ICD implantation, as long as baseline hsTnT is elevated.
- Coronary artery disease with indication for coronary revascularization/heart surgery
- Presence of intracardiac thrombi
- General contraindication for ventricular burst stimulation or intraoperative defibrillation threshold testing
- Atypical lead position requiring intraoperative defibrillation threshold testing
- Right sided device implantation
- Planned external cardioversion of atrial tachyarrhythmias
- Planned lead extraction
- Planned lead revision except for additional RV lead implantation only
- Temporary pacemaker
- Cardiogenic shock
- Pulmonary embolism, stroke, dialysis within four weeks prior to implantation
- ASA (American Society of Anesthesiologists) status 4–6 or NYHA (New York Heart Association) IV
- Inability to give or refused written informed consent,
- Age <18 years.

Patient with history of atrial fibrillation where eligible for the study when they had been on oral anticoagulation for at least four weeks prior to inclusion or a left atrial thrombus was excluded by transesophageal echocardiography prior to inclusion, if this was performed anyway during the admission.

### Study protocol

The study protocol followed a previously developed strategy^5^. Between September 2014 and June 2017 patients were randomly assigned to either (1) “ICD implantation without intraoperative antitachycardia pacing (without ATP)” or to (2) “ICD implantation with intraoperative antitachycardia pacing (with ATP)”. Intraoperative defibrillation threshold testing was not intended to be carried out in either of the groups. Random patient allocation was performed by sealed envelopes on a 1:1 basis in variable randomization blocks stratified by centre and by CRT versus non-CRT ICD systems.

### Implantation procedure

Baseline blood samples were drawn at admission or within the clinical routine to determine the baseline serum levels of hsTnT (Elecsys high sensitive Troponin T, Roche diagnostics, Rotkreuz, Switzerland), creatinkinase (total and MB fraction) and creatinine. A second determination of hsTnT was made in the operation room shortly before the beginning of the implantation procedure. All implantation procedures were performed under analgosedation with the applied drugs left to the discretion of the respective physician. Vital parameters were continuously monitored during the whole implantation procedure. A sedation level of 3 to 4 according to the Ramsay scale was intended to be maintained throughout the implantation. Following institutional standards, transvenous ICD implantation was performed placing the device either in a left-sided subpectoral or subcutaneous pocket and positioning the right ventricular lead in the right ventricular apex. Right atrial and left ventricular leads –where applicable– were also implanted according to institutional standards. After achieving adequate values for the sensing and pacing threshold of the leads, the procedure was continued according to the patient’s randomization assignment.

In patients randomized to “implantation without intraoperative antitachycardia pacing”, the pocket was closed and the patient left the operation room without further action. In patients randomized to “implantation with intraoperative antitachycardia pacing”, ventricular stimuli were administered to the myocardium in the following manner: independent of the underlying atrial rhythm (sinus rhythm or atrial arrhythmia), two ventricular burst stimulations were administered to the myocardium via the right ventricular ICD lead without prior induction of a ventricular arrhythmia. The first ventricular burst stimulation consisted of eight impulses à 6.0 volts × 1.0 ms with a cycle length of 290 ms followed by a second ventricular burst stimulation consisting of eight impulses à 6.0 volts × 1.0 ms with a cycle length of 280 ms.

To determine the postoperative serum levels of hsTnT, creatinkinase (total and MB fraction) and creatinine, a further blood sample was taken on the following morning (between14 and 20 hours after implantation).

### Procedure in case of deviations from the study protocol

All investigators were repeatedly educated to adhere strictly to the study protocol and to the randomized group. However, certain specific circumstances that could obviate the strict adherence to the testing protocol had been anticipated and recommendations for their management had been given in advance.

In cases in which the apical target region for the right ventricular lead could not be reached or yielded unsatisfactory results for the lead parameters, the implanter could select other pacing sites and it was recommended that ICD testing according to institutional standards should be performed. In the case of spontaneous appearance of ventricular or atrial arrhythmias or induction of such due to ATP application, the implanters were advised to perform ATP first and as a second step to attempt a pharmacological cardioversion (both in order to avoid shocks as far as possible). If the tachyarrhythmia persisted or if there were contraindications for pharmacological cardioversion, the implanters were free to perform an internal or external electrical cardioversion.

All deviations from the original ICD test protocol were recorded and after analysis applying the intention-to-treat principle an additional per protocol analysis was done.

### Study endpoints

The primary study endpoint was the level of myocardial micro-damage assessed by the postoperative levels of hsTnT as well as the delta in the hsTnT levels calculated from the difference between the postoperative and preoperative values.

Pre-specified secondary endpoints included the postoperative levels of the serum creatinkinase (total and MB fraction) as well as the delta in the serum creatinkinase (total and MB fraction) levels during the same observational period, and the stability of hsTnT levels calculated from the difference between preoperative and baseline hsTnT levels. All endpoints were evaluated according to the intention-to-treat principle.

### Statistics

The study is a prospective, randomized, multicenter study, aiming to compare hsTnT levels after ICD implantation with or without intraoperative rapid ventricular pacing (ATP), to assess myocardial microdamage caused by ATP. According to the earlier published TropShock study^5^, addressing myocardial microdamage after ICD shocks, a postoperative hsTnT level above 0.059 ng/ml was specified as an indicator for relevant myocardial microdamage. In the TropShock trial, a postoperative hsTnT level above 0.059 ng/ml was measured in 41% of the patients with ICD implantation only and in 67% of the patients with ICD implantation and additional shock application. It was assumed that ATP causes a similar release of hsTnT as the application of an ICD shock. Consequently, proportions of increased hsTnT levels of 41% and 67% for the two study groups were assumed based on results in the TropShock trial. Sample size was planned to obtain a power of 80% for rejection of the null hypothesis (no difference in proportions of relevantly increased postoperative hsTnT levels between groups) on a significance level of α = 0.05. This resulted in a required sample size of 128 patients (64 patients per group). Expecting an induction of ventricular and atrial tachyarrhythmias due to intraoperative ATP and consequent electrical cardioversion/defibrillation in 10% of the patients randomized to implantation with intraoperative ATP and an additional attrition rate of 10% for logistic reasons, a total of 160 patients (80 patients per group) were planned to be included in a 1:1 randomization scheme.

Assuming that the ICD implantation with intraoperative ATP compared to an implantation without ATP goes along with a 50% elevation of hsTnT based on that caused by the implantation itself^5^, this sample size is sufficiently large to detect a difference between the study groups comparing delta hsTnT (difference of postoperative and preoperative hsTnT values, power over 80%, Mann-Whitney U test).

Data analysis was performed using the software packages SPSS for Windows versions 22 and 24 (IBM Corp., Armonk, NY, USA). Analysis of the primary endpoint was carried out using the full analysis set (FAS), which was defined following the intention-to-treat principle. All patients with valid hsTnT levels were included in the analysis and each patient was analyzed in the group he/she was randomized to, irrespective of protocol deviations. The primary endpoint was additionally analyzed in the per protocol population. For categorical outcomes including the primary analysis absolute and relative frequencies are presented and group comparisons were performed using chi-squared tests. For quantitative measures, means and standard deviations for symmetrically distributed data or medians and ranges (minimum to maximum) for skewed data are shown. Two-sample t tests or Mann-Whitney U tests were used for group comparisons, as appropriate.

## Results

### Patient characteristics

Between September 2014 and June 2017, a total of 150 patients were included in the study in three centers in Germany and two centres in Switzerland. Of these 74 were randomized to “ICD implantation without intraoperative ATP” and 76 were randomized to “ICD implantation with intraoperative ATP”. Baseline patient characteristics and the procedural data are given in Tables 1 and 2 respectively. Except for left ventricular ejection fraction, baseline characteristics did not differ significantly between the two groups.

**Table 1:** Baseline characteristics.

	Total cohort (n = 150)	Without ATP (n = 74)	With ATP (n = 76)	p value
Age [years], mean ± SD	66.2 ± 11.5	67.5 ± 11.7	65.0 ± 11.2	0.083
Male gender n (%)	122 (81.3)	57 (77.0)	65 (84.2)	0.212
Ischemic cardiomyopathy n (%)	77 (51.3)	35 (47.3)	42 (55.3)	0.414
Dilated cardiomyopathy n (%)	60 (40.0)	31(41.9)	29 (38.2)	0.739
Primary prevention n (%)	140 (93.3)	68 (91.9)	72 (94.7)	0.327
CRT n (%)	45 (30.0)	21 (28.4)	24 (31.6)	0.723
LV-EF [%], mean ± SD	29.5 ± 8.7	31.3 ± 10.0	27.8 ± 6.7	0.019
Renal insufficiency n (%)	44 (30.3)	23 (23.9)	21 (28.0)	0.589
Hypertension n (%)	125 (83.3)	62 (83.8)	63 (82.9)	0.612
Diabetes mellitus n (%)	53 (35.3)	29 (39.2)	24 (31.6)	0.211
Creatinine [mg/dl], mean ± SD	1.19 ± 0.33	1.19 ± 0.32	1.18 ± 0.34	0.413
Baseline hsTnT [ng/ml], median (min-max)	0.017 (0.004–0.149)	0.018 (0.004–0.118)	0.016 (0.004–0.149)	0.947

**Table 2:** Procedural data.

	Total cohort (n = 150)	Without ATP (n = 74)	With ATP (n = 76)	p value
Subcutaneous position of ICD n (%)	95 (63.3)	50 (67.6)	45 (59.2)	0.313
Submuscular position of ICD n (%)	55 (36.6)	24 (32.4)	31 (40.8)	0.313
RV lead position Apex n (%)	124 (82.6)	63 (85.1)	61 (80.3)	0.519
RV lead position midseptal n (%)	17 (11.3)	7 (9.5)	10 (13.2)	0.608
Cut-to-suture time [min] Median (Min – Max)	57.0 (18.0–238.0)	58.0 (18.0–238.0)	56.5 (26.0–216.0)	0.103
Fluoroscopy dosis [cGycm^2^] Median (Min – Max)	119 (6–8252)	107 (8.8–1950)	125.0 (6.0–8252.0)	0.316
Contrast dye [ml] Median (Min – Max)	0 (0–100)	0 (0–100)	0 (0–100)	0.557
Intraoperative right ventricular electrode positioning [n] median (Min-Max)	2.0 (1–10)	2.0 (1–10)	1.5 (1–6)	0.846
Time ATP/Suture – hsTnT [min], Median (Min – Max)	1104 (667–2581)	1124 (769–2581)	1061 (667–1579)	0.103

### Primary endpoint

**Figure 1:**
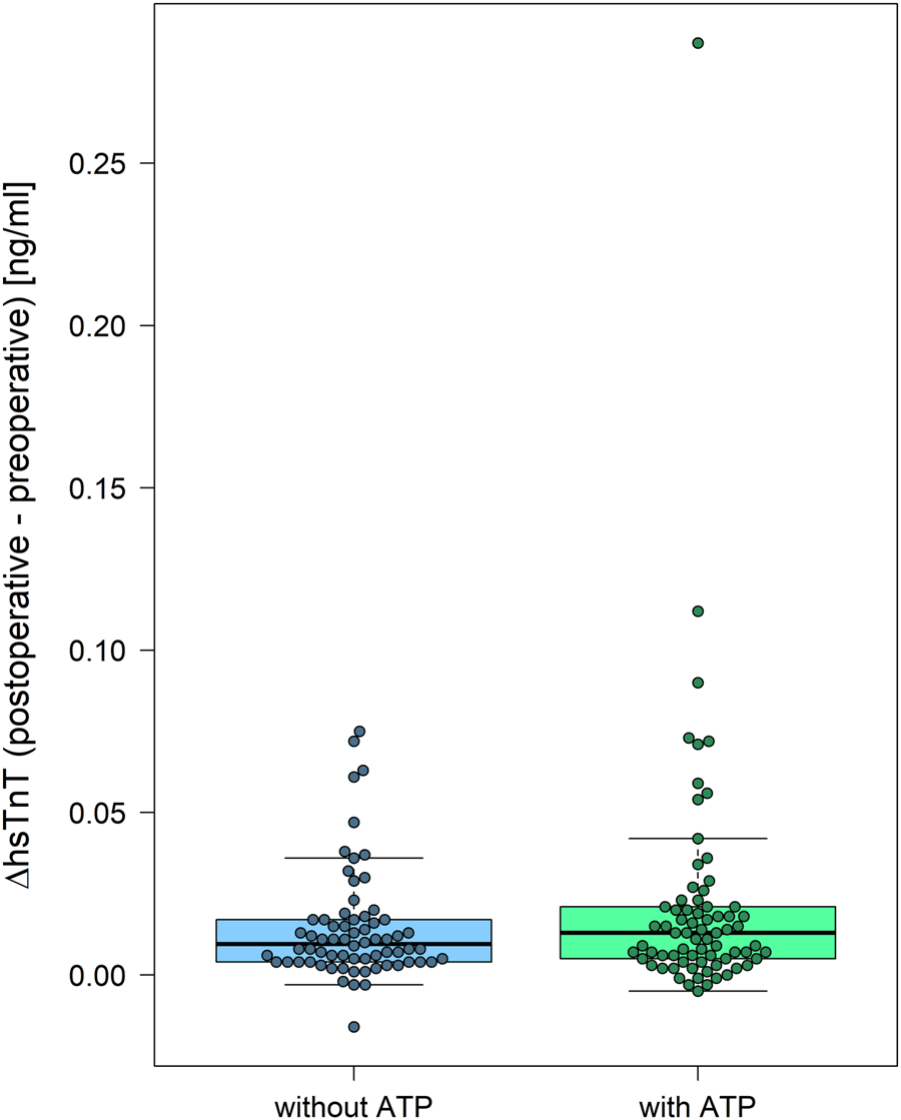
Primary endpoint; increase in hsTnT [ng/ml] (intention to treat analysis) for both randomization groups. hsTnT = high sensitive Troponin T, without ATP = implantation without antitachycardia pacing, with ATP = implantation with antitachycardia pacing.

For the primary endpoint analysis a modified intention-to-treat analysis was performed. This included 141 patients presuming that missing data of 9 patients, which had to be excluded due to missing hsTnT values for logistic reasons, was very unlikely to bias the results. For the primary analysis, a comparison of the proportion of patients with postoperative hsTnT above 0.059 ng/dl, which was defined in the study protocol as relevant myocardial microdamage, was performed. This was observed in 10 (14.7%) patients without intraoperative ATP and in 16 (21.9%) patients with intraoperative ATP (p = 0.287). Median postoperative hsTnT levels were 0.028 ng/ml (min 0.008 ng/ml – max 0.168 ng/ml) in patients without intraoperative ATP and 0.030 ng/ml (min. 0.011 ng/ml – max. 0.297 ng/ml) in patients with intraoperative ATP (p = 0.421). Considering the change in hsTnT (delta hsTnT), there was also no statistically significant difference between the two groups (no intraoperative ATP: median 0.010 ng/ml [min. −0.016 ng/ml – max. 0.075 ng/ml] versus intraoperative ATP: median 0.013 ng/ml [min. −0.005 ng/ml – max. 0.287 ng/ml], p = 0.323) (Fig. 1 and supplementary figure).

### Secondary endpoints

**Figure 2:**
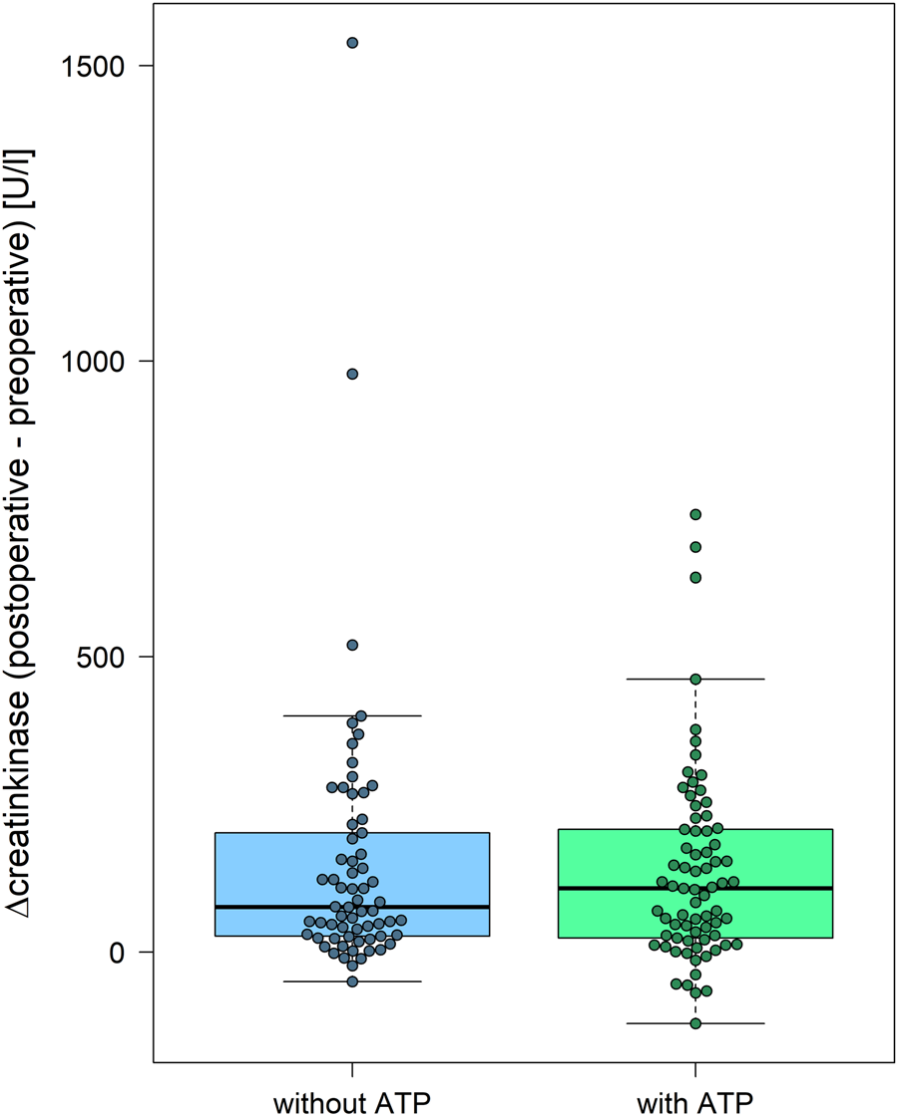
Secondary endpoint; increase in CK [U/l] (intention-to-treat analysis) for both randomization groups. CK = Creatinkinase, without ATP = implantation without antitachycardia pacing, with ATP = implantation with antitachycardia pacing.

**Figure 3:**
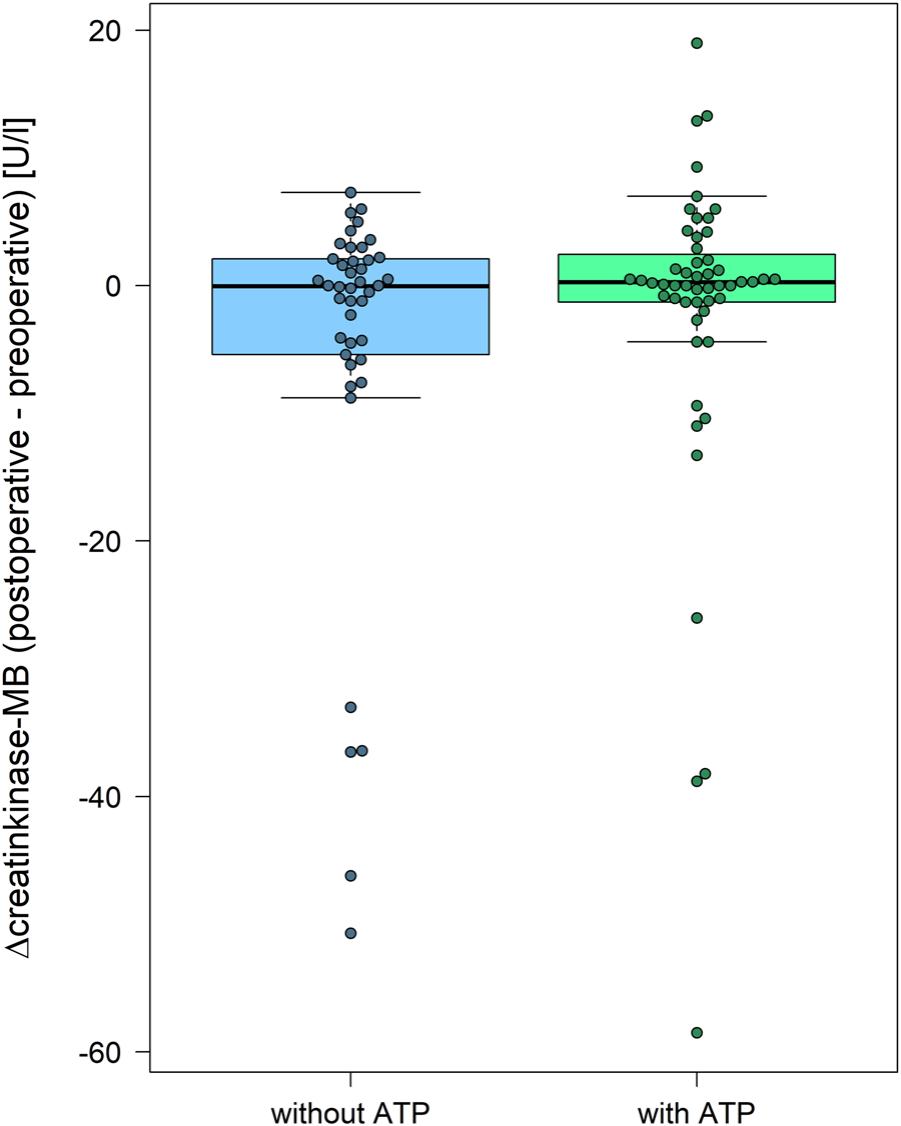
Secondary endpoint; increase in CK-MB[U/l] (intention-to-treat analysis) for both randomization groups. CK-MB = Creatinkinase MB, without ATP = implantation without antitachycardia pacing, with ATP = implantation with antitachycardia pacing.

In those patients from the full analysis set with valid CK measures (n = 138) the distribution of delta CK-levels did not differ significantly between patients without intraoperative ATP (median 76U/l [min. −50U/l – max. 1539U/l]) and the group with intraoperative ATP (108U/l [min. −121U/l – max. 740U/l]; p = 0.896) (Fig. 2). The same calculations were done for delta CK-MB-levels ((no intraoperative ATP: median −0.1U/l [min. −50.7U/l – max.7.3U/l] versus intraoperative ATP: median 0.3U/l [min. −58.5U/l – max.19U/l], p = 0.384)) and also showed no difference between the two groups (Fig. 3).

**Figure 4:**
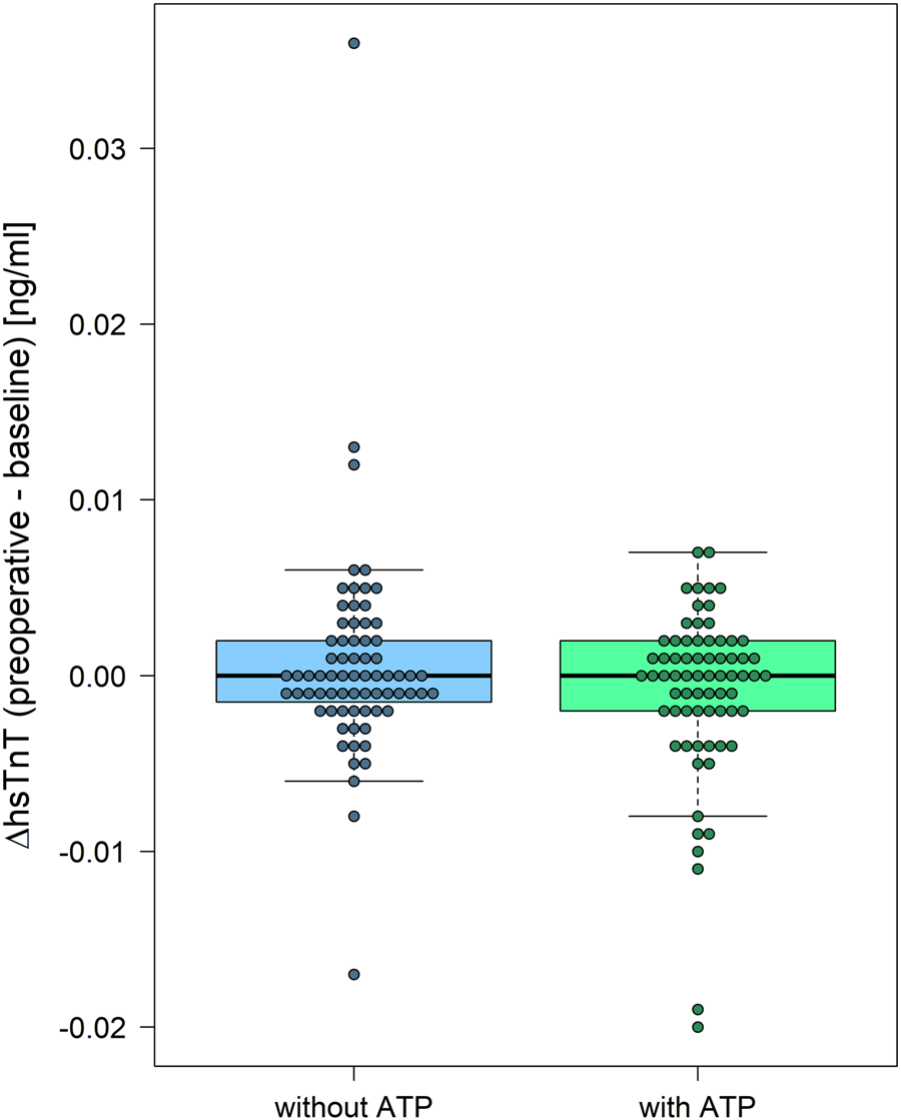
Secondary endpoint; stability of hsTnT [ng/ml] (intention to treat analysis) for both randomization groups. hsTnT = high sensitive Troponin T, without ATP = implantation without antitachycardia pacing, with ATP = implantation with antitachycardia pacing.

To prove stability of hsTnT levels over time, delta hsTnT was calculated from the difference between preoperative and baseline hsTnT levels, which were taken on admission. These delta hsTnT levels showed stability of hsTnT between the baseline levels at admission and the preoperative determination of hsTnT in both groups (no intraoperative ATP: delta hsTnT median 0.000 ng/ml [min. −0.017 ng/ml – max. 0.036 ng/ml] versus intraoperative ATP: delta hsTnT median 0.000 ng/ml [min. −0.020 ng/ml – max. 0.070 ng/ml], p = 0.063) (Fig. 4 and supplementary figure).

### Per-protocol analysis

In 6 patients randomized to “ICD without intraoperative ATP (no ATP)” and in 4 patients randomized to “ICD with intraoperative ATP (ATP)” the study protocol could not be performed as intended. The main reasons for non-adherence to the protocol were missing hsTnT measurements (6 patients in the “no ATP” group and 3 patients in the “ATP” group), as mentioned above. Moreover, in the “ATP” group, there was one patient, in whom sustained ventricular tachycardia was induced by intraoperative ATP which was terminated by internal shock (42 J). The remaining 140 patients were treated according to the protocol and were included in the per protocol analysis.

For this cohort the per-protocol-analysis showed similar results as earlier described above for the intention-to-treat population. There was no significant difference in postoperative absolute and delta hsTnT levels in both groups, as well as no significant difference in the delta CK and CK-MB levels.

### Induction of arrhythmias caused by ATP

Neither the acceleration nor the induction of any atrial arrhythmia was caused by ATP in the intervention group. Sustained monomorphic ventricular tachycardia was induced by ATP in 3 patients in the “ATP” group (3,9%). In two of these patients ventricular tachycardia was terminated by ATP. In one patient the ventricular tachycardia had to be terminated by internal ICD shock (42 Joule) due to its short cycle length (285 ms; 210bpm) and consecutive hemodynamic instability.

## Discussion

The aim of the MyDate study was to define myocardial microdamage caused by antitachycardia pacing. The main findings of the study are: (1) there is no significant difference in the post-operative rise of hsTnT levels between a group of patients randomized to “ICD implantation without intraoperative ATP” and a group of patients randomized to “ICD implantation with intraoperative ATP”. (2) hsTnT levels show reliable stability over time in the study population. (3) the induction of sustained ventricular tachycardia due to ATP is rare.

Data concerning myocardial microdamage caused by antitachycardia pacing is scarce. In earlier days of ICD therapy a non randomized study showed no relevant myocardial injury after pacing terminated ventricular tachycardia in 10 patients, but the group of patients was small, a ventricular arrhythmia was induced prior to ATP and laboratory parameters refered to Troponin T but not to hsTnT, which today is commonly used to assess myocardial micro-damage^6^. Recently available data of prospective randomized trials only refer to determination of cardiac enzymes after ICD implantation and additional ICD shocks^4,5^. The results of these trials suggest, that there is myocardial microdamage, not only caused by the implantation of the ICD but also related to ICD shocks themselves. In a subanalysis of the recently published randomized SIMPLE trial, postoperatively elevated troponin levels after ICD implantation lead to an unfavorable longterm outcome namely an increased all-cause mortality and a higher risk of arrhythmic death^4^. In patients with additional intraoperative defibrillation threshold testing, elevated troponin levels were more commonly detected than in patients without defibrillation threshold testing. In other entities, such as heart failure, acute coronary syndroms, stable coronary artery disease and atrial fibrillation, elevated levels of Troponin have not only been shown to be associated with myocardial microdamage but also with increased mortality and morbidity^7–9^. By randomizing patients either to an ICD implantation without intraoperative ATP or to an ICD implantation with intraoperative ATP, we aimed to characterize myocardial microdamage caused by ATP. In this study population we found no difference in postoperative levels of cardiac enzymes between the two randomization groups, suggesting that there is no relevant myocardial damage due to ATP. Moreover, we proved in a secondary analysis that the preoperative hsTnT levels were stable over time and thus excluded the possibility of unstable measurements influencing the results. However, because our trial was designed as an acute study, we are not able to conclude on potential long-term effects of ATP on morbidity and mortality. The use of ATP is well established in ICD therapy, being known for its efficient and painfree termination of slow as well as fast ventricular tachycardias, while reducing ICD shocks and patient discomfort at the same time^1,2,10–13^. However, the ongoing international discussion of whether or not ICD therapies themselves lead to an increase in mortality expanded from the initially targeted ICD shocks to ATP. In 2012 the prospective randomized MADIT-RIT study, designed to compare different ICD programming strategies to reduce the number of inadequate ICD therapies, suggested that a significant reduction of appropriate and inappropriate ATP leads to a relevant reduction of mortality and raised the question of whether ATP itself was responsible for a worse outcome^3^. Since then, several studies sought to define the impact of ATP on mortality. In a prospective single-center registry including almost 1400 patients, ATP as first therapy after implantation was associated with an adverse prognosis and was followed by further ATP or ICD shocks in the clinical course, hence suggesting a progression of the underlying heart disease^14^. Strickberger *et al*. analyzed remote-monitoring data of nearly 70000 patients and found an improved survival in patients receiving ATP versus patients receiving ICD shocks for an underlying ventricular arrhythmia, but there was still an increased mortality risk compared to patients without any ICD therapy. In this real-world population, the success rate of ATP in the termination of ventricular tachycardia was >85%, confirming its efficiency^15^. Recently, a prospective multicenter observation study including 1404 patients found no association of adequate or inadequate ATP and mortality^11^ and goes along with many observational and randomized trials earlier showing no impact of ATP on mortality either^2,16–20^. Meanwhile a subanalysis of the MADIT-RIT study identified adequate shocks, inadequate ICD therapies and a conventional aggressive ICD programming as independent predictors of mortality. Appropriate ATP itself was no relevant independent predictor of mortality in this subanalysis^21^. Whether ATP itself or the progression of the underlying heart disease leading to ventricular arrhythmias is associated with an increase in mortality still remains unclear. To answer this question, randomized studies exclusively addressing this topic are warranted in the future.

The concern that inadequate ATP might induce or accelerate arrhythmias and therefore might lead to additional shock applications was addressed in a few studies, showing that the risk of induction of supraventricular and ventricular arrhythmias is generally very low^11,18,22^, whereas the acceleration of ventricular arrhythmias range between 1.5% and 28%^2,23,24^. Our findings conform with these studies, documenting three sustained ventricular tachycardias induced by intraoperative ATP in three different patients (3.9%). Only one of these had to be treated by internal cardioversion due to its short cycle length, two others were successfully treated by ATP. No supraventricular tachycardia was induced or accelerated in our population.

Because our trial was designed as an acute study we are not able to conclude on potential long-term effects of ATP on morbidity and mortality. However, our findings, that there is (1) no relevant myocardial microdamage caused by ATP in this study population and (2) ventricular tachycardias induced by ATP are rare, may support its use as a painless and efficient method to terminate ventricular tachycardia in high-risk patients.

### Limitations

The study was designed to determine potential myocardial micro-damage caused by intraoperative ATP. To apply the same ATP energy levels and cycle lengths on the myocardium the devices of only one manufacturer were implanted. We therefore cannot make a conclusion about ATP applied by other manufacturers. Moreover, no conclusion can be made about other types of rapid ventricular pacing. The manufacturer of the leads was left to the physicians’ choice. As all implanted leads in this study were active fixation leads, we are not able to comment about myocardial micro-damage caused by ATP and passive fixation leads. However, in a recent study, the type of pacemaker lead fixation (active versus passive fixation leads) did not significantly influence the extent of myocardial injury^25^. Due to ethical and logistical reasons, postoperative blood samples were taken the next morning. This might have led to a determination of hsTnT outside its peak. However, the time from intraoperative ATP/suture to determination of hsTnT was not significantly different in both randomization groups. This study was not created in a dose-response manner and can therefore not give evidence in this point. Moreover, as it was designed to determine acute effects of ATP on myocardial damage, no conclusion can be made about potential longterm effects.

## Conclusion

Additional intraoperative ATP did not lead to a significant rise in hsTnT release compared to an ICD implantation without ATP. Hence, antitachycardia pacing seems not to cause relevant myocardial microdamage. This may further support its use as a painfree and effective method to terminate ventricular tachycardia in high-risk patients and may provide proponents for a delayed ICD therapy, favoring ATP, with additional arguments.

## Supplementary information

Supplementary information

Supplementary information2

## Data Availability

The datasets generated and analysed during the current study are available from the corresponding author on reasonable request.
