# Supplementary material for: Myocardial Minimal Damage After Rapid Ventricular Pacing – the prospective randomized multicentre MyDate-Trial
Source: Sci Rep. 2020 Mar 16;10:4753. doi: 10.1038/s41598-020-61625-8 (PMC7075963; doi:10.1038/s41598-020-61625-8)
Supplement: Supplementary file 2 — Supplementary information2 [file 41598_2020_61625_MOESM2_ESM.pdf]

Myocardial Minimal Damage After Rapid Ventricular Pacing – the prospective randomized multicentre MyDate-Trial

Verena Semmler[1], Clara Deutschmann[1], Bernhard Haller[2], Carsten Lennerz[1,3], Amir Brkic[1], Christian Grebmer[1], Patrick Blazek[1], Severin Weigand[1], Martin Karch[4], Sonia Busch[5], Christof Kolb[1]

1) Deutsches Herzzentrum München, Klinik für Herz- und Kreislauferkrankungen, Abteilung Elektrophysiologie, Technische Universität München, Munich, Germany

2) Klinikum rechts der Isar, Institut für Medizinische Informatik, Statistik und Epidemiologie, Fakultät für Medizin, Technische Universität München, Munich, Germany

3) DZHK (German Centre for Cardiovascular Research), partner site Munich Heart Alliance, Munich, Germany

4) Herz- und Gefäßzentrum Oberallgäu-Kempten, Klinikum Kempten, Kempten, Germany

5) Klinikum Coburg, Abteilung für Kardiologie und Angiologie, Coburg, Germany

Address for correspondence: Dr. med. Verena Semmler
Deutsches Herzzentrum München
Lazarettstr. 36
80636 München
Germany
Phone 0049 89 1218 2020
Fax 0049 89 1218 4593
E-mail: semmler@dhm.mhn.de

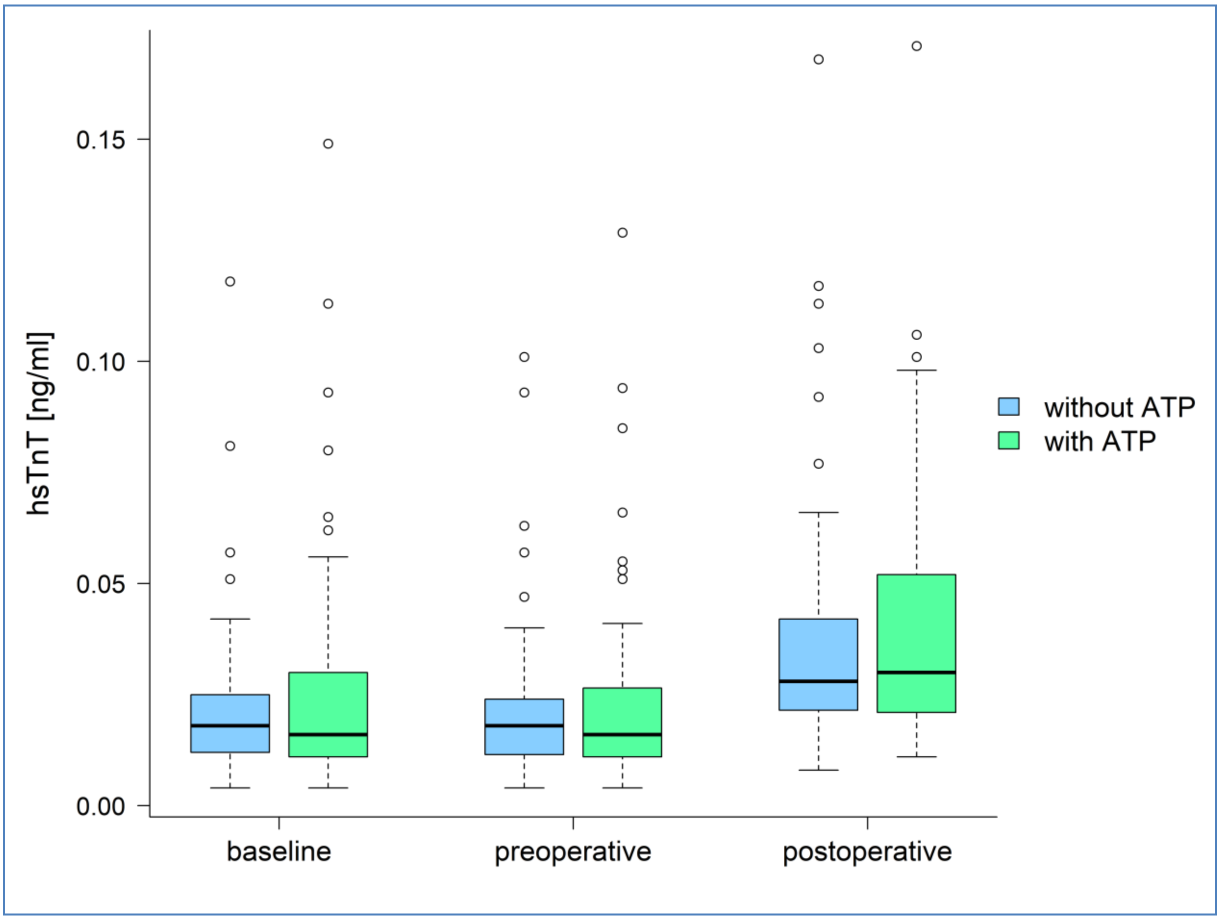

Supplementary figure. Absolute hsTnT [ng/ml] values (intention to treat analysis) for both randomization groups at baseline, preoperative and postoperative. hsTnT = high sensitive Troponin T, without ATP = implantation without antitachycardia pacing, with ATP = implantation with antitachycardia pacing
